# AI‐based intra‐tumor heterogeneity score of Ki67 expression as a prognostic marker for early‐stage ER+/HER2− breast cancer

**DOI:** 10.1002/cjp2.346

**Published:** 2023-10-24

**Authors:** Wenqi Lu, Ayat G Lashen, Noorul Wahab, Islam M Miligy, Mostafa Jahanifar, Michael Toss, Simon Graham, Mohsin Bilal, Abhir Bhalerao, Nehal M Atallah, Shorouk Makhlouf, Asmaa Y Ibrahim, David Snead, Fayyaz Minhas, Shan E Ahmed Raza, Emad Rakha, Nasir Rajpoot

**Affiliations:** ^1^ Tissue Image Analytics (TIA) Centre, Department of Computer Science University of Warwick Coventry UK; ^2^ Academic Unit for Translational Medical Sciences, School of Medicine University of Nottingham Nottingham UK; ^3^ Department of Pathology, Faculty of Medicine Menoufia University Menoufia Egypt; ^4^ Department of Pathology University Hospitals Coventry and Warwickshire NHS Trust Coventry UK

**Keywords:** AI based prognostic biomarkers, computational pathology, Ki67 expression, luminal breast cancer

## Abstract

Early‐stage estrogen receptor positive and human epidermal growth factor receptor negative (ER+/HER2−) luminal breast cancer (BC) is quite heterogeneous and accounts for about 70% of all BCs. Ki67 is a proliferation marker that has a significant prognostic value in luminal BC despite the challenges in its assessment. There is increasing evidence that spatial colocalization, which measures the evenness of different types of cells, is clinically important in several types of cancer. However, reproducible quantification of intra‐tumor spatial heterogeneity remains largely unexplored. We propose an automated pipeline for prognostication of luminal BC based on the analysis of spatial distribution of Ki67 expression in tumor cells using a large well‐characterized cohort (*n* = 2,081). The proposed Ki67 colocalization (Ki67CL) score can stratify ER+/HER2− BC patients with high significance in terms of BC‐specific survival (*p* < 0.00001) and distant metastasis‐free survival (*p* = 0.0048). Ki67CL score is shown to be highly significant compared with the standard Ki67 index. In addition, we show that the proposed Ki67CL score can help identify luminal BC patients who can potentially benefit from adjuvant chemotherapy.

## Introduction

Breast cancer (BC) is the most commonly diagnosed cancer among women and the second leading cause of cancer death in females worldwide [1]. Although estrogen receptor (ER) negative and human epidermal growth factor receptor (HER2) positive BC are associated with poor prognosis, the luminal (ER+/HER2−) tumors, which account for about 70% of all BCs [2], are clinically indeterminate. Their biology, patient outcome, and clinicopathological characteristics make luminal BCs quite heterogeneous tumors and more challenging in terms of treatment decisions [3]. Although adjuvant endocrine therapy is the mainstay of systemic treatment for patients with luminal BC, some of them may also benefit from the addition of cytotoxic chemotherapy [4]. Therefore, risk stratification of this subtype to guide the use of chemotherapy is vital.

Several gene expression profile‐based tests have been developed to identify patients who may benefit from chemotherapy [5, 6]. However, these tests are expensive and may incur long turnaround times [5, 7]. Image‐based techniques have the advantages of standardization and saving time and cost [8]. Therefore, developing alternative methods, such as image‐based prognostic indicators, to avoid over‐ and under‐treatment of patients in this intermediate risk group needs to be explored.

Ki67, as a biomarker of cellular proliferation, has been shown to be prognostic of clinical outcome in early‐stage luminal BC [9] and a predictor of response to neoadjuvant chemotherapy [10]. However, Ki67 scoring shows poor reproducibility due to lack of standardization and subjectivity of its assessment. The national Swedish guidelines state that, in a Ki67 hot‐spot region, 200 tumor cells should be counted with notation of the number of Ki67‐positive tumor cells as a percentage [11]. In contrast, the International Ki67 Breast Cancer Working Group recommended that at least 1,000 cells should be counted with 500 cells accepted in representative fields as the absolute minimum [12]. Other methods of Ki67 scoring include subjective evaluation of the percentage of positive cells in the hot‐spot area without determination of the cell count within such areas [13]. Automated Ki67 scoring methods, including digital image analysis (DIA) [14], have been proposed for improving the reproducibility of Ki67 scoring. However, the prognostic significance of these automated Ki67 scoring methods can be improved further and methods involving new features along with existing Ki67 scoring need to be explored.

Various studies have shown that the spatial colocalization of different types of cell is clinically important in different cancers. Galon *et al* [15] found that the immunological data (i.e. type, density, and location of immune cells within the tumor samples) are good predictors of patient survival in colorectal cancer. Shaban *et al* [16] proposed a digital score for abundance of tumor‐infiltrating lymphocytes to predict disease‐free survival in oral squamous cell carcinoma. In addition, Maley *et al* [17] revealed that measuring spatial colocalization of immune and cancer cells can be a prognostic BC biomarker for BC. However, most of the studies investigate the spatial organization of immune cells in relation to tumor cells but colocalization patterns of Ki67‐positive and Ki67‐negative tumor remain largely unexplored. With the increasing use of digital pathology and artificial intelligence (AI) algorithms it is now possible to refine the scoring of immunohistochemistry (IHC) markers such as Ki67 and consider not only digital scoring but also other features including spatial distribution.

Ki67 expression shows a high degree of heterogeneity, which influences tumor behavior and patient outcome [13]; with accurate cell detection and classification this heterogeneity can be captured spatially. The importance of spatial distribution of Ki67 has recently been investigated to find a more reproducible and less subjective way of quantification. For example, DIA of Ki67 in hot‐spots was found to be superior to both manual Ki67 and mitotic counts [18]. Similarly, the prognostic ability of Ki67 hot‐spot scoring was found to be better or comparable to average scoring in whole slide images (WSIs) though the hot‐spot scoring may vary to some extent with the selection of the hot‐spots [18, 19].

To address these challenges, we propose an AI‐based risk stratification model to automatically generate the Ki67 colocalization (Ki67CL) score, which measures the spatial colocalization between Ki67‐positive and Ki67‐negative tumor cells. It is based on the hypothesis that less even distribution of Ki67 tumor cells could enable risk stratification of ER+/HER2− BC patients and behave as an alternative to the expensive gene expression profiling assays. It should be noted that stratification of this intermediate category of BC (i.e. ER+/HER2−) is more challenging because of its clinically low risk of recurrence. The aim of this study is to develop a robust prognostic tool using Ki67 to stratify this intermediate group of patients who are treated only with endocrine therapy into distinct risk groups, reducing the need for aggressive therapies for low‐risk patients, and preventing the undertreatment of high‐risk patients. We report the application of our proposed tool on a large dataset (*n* = 2,081 patients) and show that, in comparison to Ki67 scoring, the Ki67CL score can risk stratify luminal BC patients more significantly and adds more information above other clinicopathological features.

## Materials and methods

This study was approved by the Yorkshire & The Humber – Leeds East Research Ethics Committee (REC Reference: 19/YH/0293) under the IRAS Project ID: 266925. The data collected were fully anonymized.

### Dataset description

The dataset used in this study was obtained from Nottingham University Hospital, Nottingham, UK. Our study cohort consists of 2,172 ER+/HER2− BC patients with 0–3 positive lymph nodes (LN 0–3) and with more than 10 years of follow up. Ninety‐one cases were excluded during the quality control process because of the presence of excessive tissue folding, large blurry regions, or no tumor tissue being detected. Among the remaining 2,081 cases included in this study, 1,968 patients received endocrine therapy without chemotherapy while 113 patients who received both endocrine therapy and chemotherapy were treated as a control group. The cases were divided into discovery/validation (*n* = 1,393, 67%) and test (*n* = 685, 33%) sets. The model was developed on the discovery/validation set by splitting this set into three folds. Supplementary material, Table S1 lists the number of cases in discovery and validation in the three splits. Tissue samples from resections (Ki67‐stained slides) were scanned at ×40 magnification with two different scanners (Philips IntelliSite Ultrafast Scanner, Philips Digital Pathology Solutions, Best, The Netherlands and Pannoramic 250 Flash III, 3DHISTECH, Budapest, Hungary). For each case, the dataset contains a set of clinicopathological parameters such as patient's age at diagnosis, tumor grade, invasive tumor size and type, lymphovascular invasion (LVI), LN status, and Nottingham prognostic index (NPI). Outcome data, including BC‐specific survival (BCSS) and distant metastasis free survival (DMFS) were collected for all patients. BCSS was defined as the time (in months) from diagnosis to the last date that the patient was known to be alive, lost to follow‐up, or death. Patients who died from unrelated causes were censored while death from BC was considered as an event. BC‐related death was defined as death from or with metastatic BC as stated in the postmortem report or death certificate (e.g. metastatic disease and/or its complications). DMFS was defined as the time (in months) from diagnosis until the first event of distant metastasis. For each patient, one Ki67‐stained WSI was available for developing an image‐based machine learning (ML) method for survival prediction.

### Dataset preparation

Full‐face sections were prepared from the selected formalin‐fixed paraffin‐embedded tissue blocks. The IHC staining was performed automatically using the clinically validated DAKO Cytomation EnVision+ detection system according to standard protocols [20]. The Ki67‐stained slides were scanned using two high‐resolution scanners i.e. Philips Ultrafast Scanner and Pannoramic 3DHISTECH Scanner to scan about 1,376 and 705 cases, respectively. The scanned slides were then uploaded to our WASABI server (a customized version of HistomicsTK [21]), which is used for marking annotations and visualizing WSIs.

Annotations were marked at region level and cell level to develop ML models for region segmentation and cell detection and classification. Region annotations included tumor, ductal carcinoma *in situ* (DCIS), and normal regions while cell annotation included Ki67‐positive tumor, Ki67‐negative tumor, positive nontumor, and negative nontumor cells. In total, we collected annotations for around 600 mm^2^ of tumor area, 140 mm^2^ of normal area, 380 mm^2^ of DCIS area, and 9,433, 26,308, 2,424, 19,223 of the four cell types, respectively.

### Computational framework

**Figure 1:**
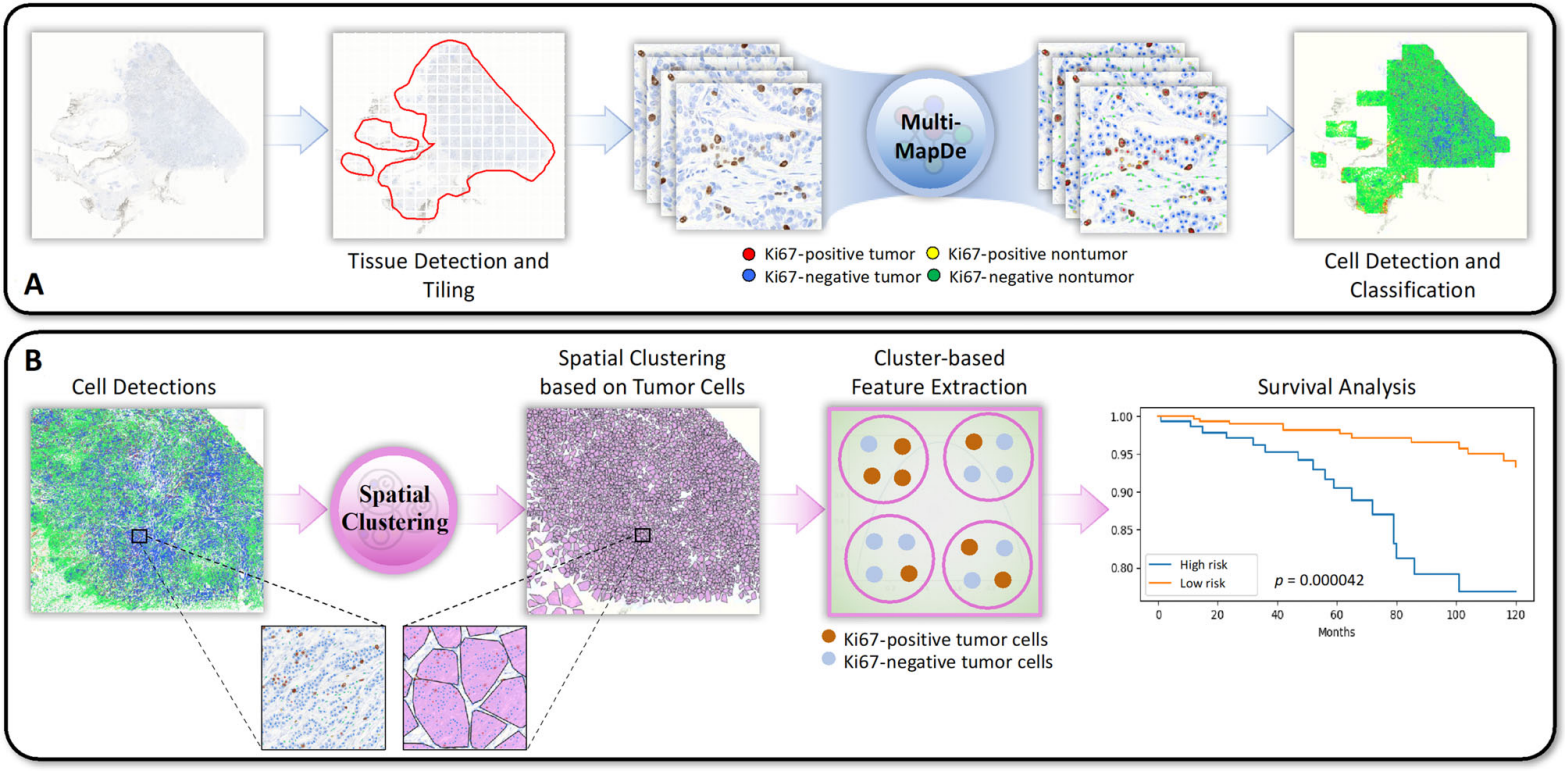
Workflow of the proposed AI pipeline for stratifying high‐ and low‐risk luminal BC patients. (A) Cells are classified as Ki67‐positive or Ki67‐negative tumor cells, and Ki67‐positive or Ki67‐negative nontumor cells in the detected tissue area. (B) Colocalization of Ki67‐positive and Ki67‐negative tumor cells is measured following spatial clustering and used for survival analysis.

Figure 1 shows the overall computational framework, which consists of four main components: simultaneous cell detection and classification, spatial clustering based on Ki67 expression in tumor cells, feature extraction, and survival analysis. First, a simultaneous cell detection and classification network was developed to detect four different kinds of cell (supplementary material, Figure S1), including Ki67‐positive tumor cells, Ki67‐negative tumor cells, positive nontumor cells, and negative nontumor cells, based on pathologists' annotations. Second, we used spatial clustering to group spatially neighboring tumor nuclei into clusters in which Ki67 expression was measured. Third, spatial colocalization (evenness) between Ki67‐positive and Ki67‐negative tumor cells (Ki67CL score) was used to quantify the diversity of Ki67 tumor cells in a tissue slide. The power of the Ki67CL score was evaluated against the automated Ki67 score. Finally, the prognostic significance of Ki67CL score in terms of BCSS and DMFS was evaluated by employing univariate and multivariate analyses including other clinical prognostic factors.

### Simultaneous cell detection and classification network

We developed and trained a deep learning (DL) neural network model to classify cells in input images into four types. Training a DL network on entire WSIs at full resolution is computationally intractable as the size of WSIs at the highest resolution can be up to 150,000 × 100,000 pixels. Therefore, we removed the background areas and divided the tissue region into small regions (patches) of size 252 × 252 pixels at ×40 and each patch was processed independently by the DL model to identify relevant cells. The model was trained for 30 epochs with a learning rate of 0.0001 on patches from 20 WSIs and tested on 12 WSIs. All the training and validation experiments were performed on a Dell Precision 5820 Tower Workstation, Dell Technologies, Round Rock, Texas, USA equipped with one NVIDIA T400 GPU. In total, 3,000 patches were annotated by a group of six medically qualified pathologists (Ayat Lashen, Islam Miligy, Michael Toss, Asmaa Ibrahim, Ayaka Katayama, and Henry Ebili).

To measure inter‐observer agreement, 300 patches were randomly selected and were independently annotated as dots by two pathologists (Ayat Lashen and Islam Miligy) for the four types of cell where a good agreement (Kappa score = 0.85) was reached. Annotations for which the centroids of the dots were within 20 pixels were counted as the same cell annotation. The same criteria were used when evaluating ML model predictions against the ground truth.

**Figure 2:**
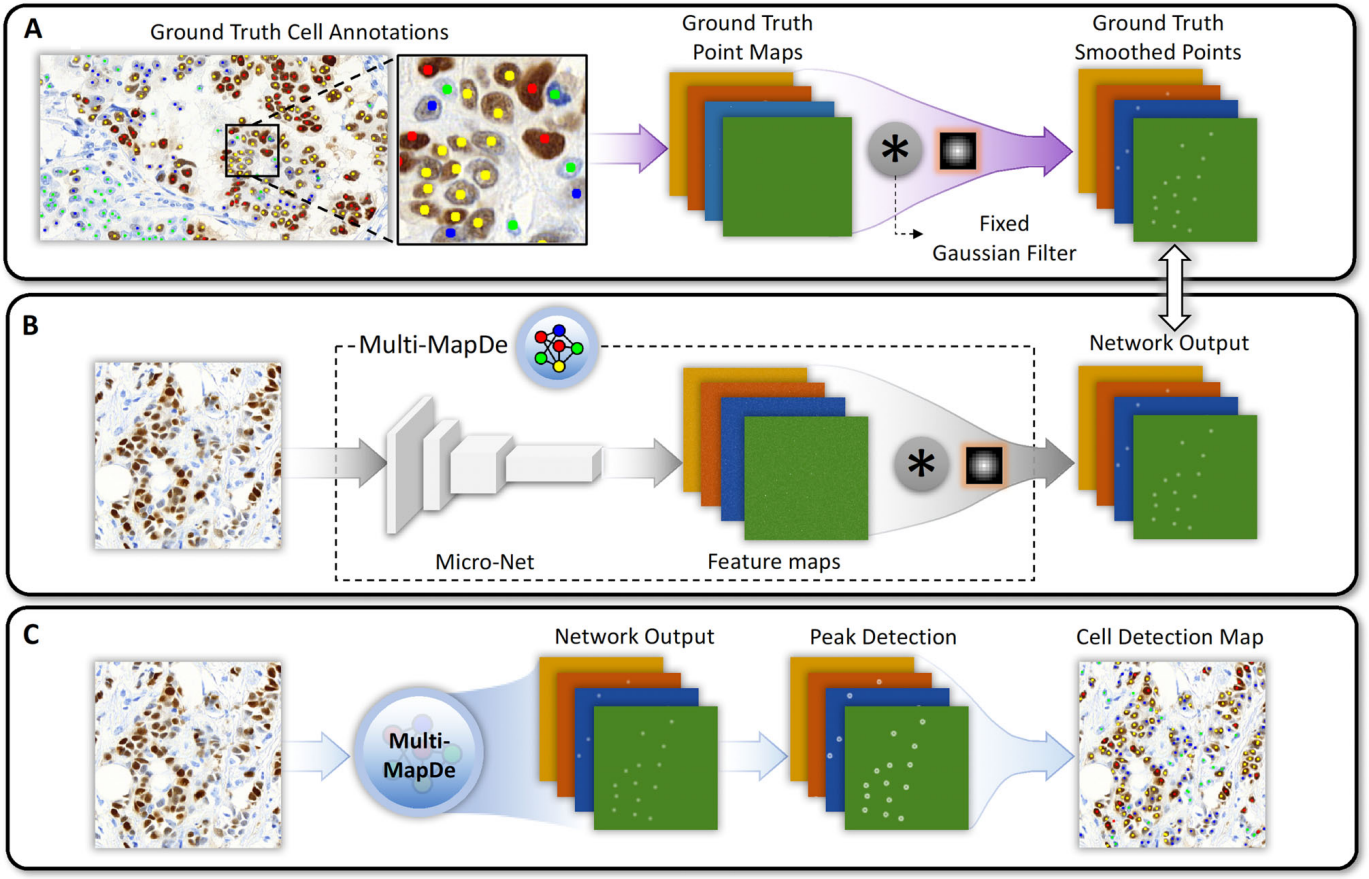
Overview of the Multi‐MapDe method. (A) Preprocessing. The dot annotations at each class channel are convolved with a Gaussian filter to generate artificial labels. (B) Training. The final layer of a multi‐output Micro‐Net is convolved with the same (not trainable) mapping filter. The network output is trained with the artificial labels from step (A). (C) Inference. For a new image, the output of the trained network is a multichannel image in which the number of channels is same to the number of cell classes.

The proposed simultaneous cell detection and classification network (Multi‐MapDe) is a significant enhancement of a previously proposed cell detection network (MapDe) [22] and employs an extended network architecture that enables the model to detect all cell types simultaneously. The overview of the proposed Multi‐MapDe is illustrated in Figure 2. First, pathologists' annotations were translated to multichannel dot labels where each channel represented one cell type. The binary dot labels were convolved with a Gaussian filter to generate artificially mapped labels. Second, input images were fed into the Micro‐Net [23] architecture and the output layer of Micro‐Net was convolved with the same mapping filter. The modified network was trained to learn the artificial labels from the first step. Third, for the probability map at each output channel, we regressed the probability of belonging to the center of a cell followed by detecting its local maxima. Last, we merged detected local maxima in all channels. For multiple local maxima that were less than 15 pixels apart, we only kept the one with the highest probability.

### Proposed prognostic marker

We proposed an ML‐based automated score, called Ki67CL score, to measure the spatial colocalization between Ki67‐positive tumor and Ki67‐negative tumor cells. It is based on the hypothesis that high diversity of Ki67 tumor cells could enable risk stratification of ER+/HER2− BC patients. The CL score was calculated in two steps: spatial clustering based on tumor cells in the WSI regions and calculation of the colocalization statistics.

In the first step, given the predicted tumor cells from the cell detection and classification stage, we used agglomerative clustering [24] to group spatially neighboring tumor cells into clusters. Specifically, we selected a random subset of 10,000 tumor cells for agglomerative clustering using the Euclidean distance metric with average linkage. Using the discovery set, a distance threshold of 300 pixels for cluster agglomeration was selected empirically by analyzing the performance on survival prediction with different distances. This resulted in a set of clusters with each cell assigned to exactly one cluster. We then used the nearest neighbor rule [24] to assign all remaining tumor cells to neighboring clusters.

In the second step, colocalization statistics were calculated at each cluster and then averaged to obtain the WSI‐level Ki67CL score. Here we used the Shannon diversity index [25] to quantify the colocalization of two cell classes in a given cluster. The proposed Ki67CL score is defined using the following formula:

(1) $$\mathrm{Ki}67\mathrm{CL}\;\text{score}=-\frac{1}{C}\sum_{i=1}^{C}p_{i}^{+}\log_{2}\left(p_{i}^{+}\right)+\left(1-p_{i}^{+}\right)\log_{2}\left({1-p}_{i}^{+}\right),$$

where $p_{i}^{+}$ is the percentage of Ki67‐positive tumor cells in the cluster *i*, $\left({1-p}_{i}^{+}\right)$ is the percentage of the corresponding Ki67‐negative tumor cells, and C is the total number of clusters. Mathematically, the Ki67CL score ranges from 0 to 1. It achieves a peak value when both cell types have the same proportion in each cluster. If all the clusters contain only one cell type either Ki67 positive or negative, the colocalization score becomes zero, as shown in supplementary material, Figure S2.

To compare our proposed Ki67CL score with automated Ki67 score, we used the same pipeline as discussed above to identify the cell types but calculated the automated Ki67 (AutoKi67) ratio using the percentage of predicted Ki67‐positive tumor cells in the invasive tumor region.

### Survival analysis

To estimate the prognostic significance of the proposed Ki67CL score, survival analysis was performed in Python using the Kaplan–Meier model and a log‐rank test to stratify patients into low‐risk (long‐term survival) and high‐risk (short‐term survival) groups. In order to find the optimal cutoff point, three‐fold cross‐validation was carried out on the modeling subset and the corresponding *p* value was reported. The optimal cutoff point (Ki67CL score: 0.375, AutoKi67 ratio: 0.177) was then evaluated on the final test set. We used concordance index (*C*‐index) to measure the rank correlation between predicted risk scores and patients' survival time. In the three‐fold cross‐validation, we reported a ‘combined *p* value’ and a ‘combined *C*‐index’, where the former is calculated by two times the median of *p* values to get a conservative estimate [26] from three folds, while the latter is calculated by taking the mean and standard deviation of the *C*‐index from each fold.

The proposed Ki67CL score was compared with the widely used Ki67 ratio (AutoKi67 ratio), which is calculated using the percentage of Ki67‐positive tumor cells in the invasive tumor region. AutoKi67 ratio is normalized between 0 and 1, and the same cutoff selection strategy is used in AutoKi67 ratio analysis as is used for the Ki67CL score. The Cox proportional hazard regression model was used for univariate and multivariate analyses. We report the hazard ratio along with lower and upper 95% confidence interval (CI). Several features including clinical factors and proposed digital marker were evaluated simultaneously in multivariate analysis. In addition, we investigated the influence of inclusion of DCIS regions in this analysis. We extracted both features (Ki67CL score and AutoKi67 ratio) from two settings: the WSI level (with DCIS) and the invasive tumor region level (without DCIS), and evaluated their prognostic performance and stability.

## Results

### Patient and tumor characteristics

The mean age of the patients at diagnosis was 60 years with a range of 27–90. Tumor size ranged from 0.15 to 10 cm with a mean of 1.69 cm. Approximately, 22%, 57%, and 21% of the patients had Nottingham Histological Grade 1, 2, and 3 tumors, respectively. Seventy‐eight percentage of the patients had LN‐negative tumors whereas 22% had 1–3 positive LNs. Supplementary material, Table S2 lists detailed patient and tumor characteristics.

### Cell detection and classification

Performance of the cell detection method was evaluated against the ground truth in terms of different metrics. For all the cell classes the Kappa score was 0.76, Spearman correlation score was 0.87 with a highly significant *p* value of 1.88 × 10^−238^, accuracy was 0.91, and F1‐score was 0.81. Classification results for individual cell classes are listed in supplementary material, Table S3. Example visual results for cell detection and classification are presented in supplementary material, Figure S3 which shows the accurate classification capability of the Multi‐MapDe pipeline.

The Ki67CL score was *μ* = 0.377, *σ* = 0.118 while the AutoKi67 ratio was *μ* = 0.220, *σ* = 0.114. In total, 695 (51%) patients had a low Ki67CL score while 692 (49%) had a high Ki67CL score. Similarly, for AutoKi67 ratio, 561 (40%) and 826 (60%) had low and high scores. High Ki67CL score showed a significant association with high histologic tumor grade (*p* < 0.0001), poor NPI (*p* < 0.0001), and LVI (*p* = 0.009) for one‐sided Mann–Whitney *U* test (supplementary material, Table S4). Similarly, AutoKi67 ratio showed association with high histologic tumor grade (*p* < 0.0001) and poor NPI (*p* < 0.0001) but a no significant association with LVI (*p* = 0.8322). However, looking at the *p* values in both analyses, higher significance was found using Ki67CL score.

### Outcome analysis

#### Survival performance on the validation and testing sets

**Figure 3:**
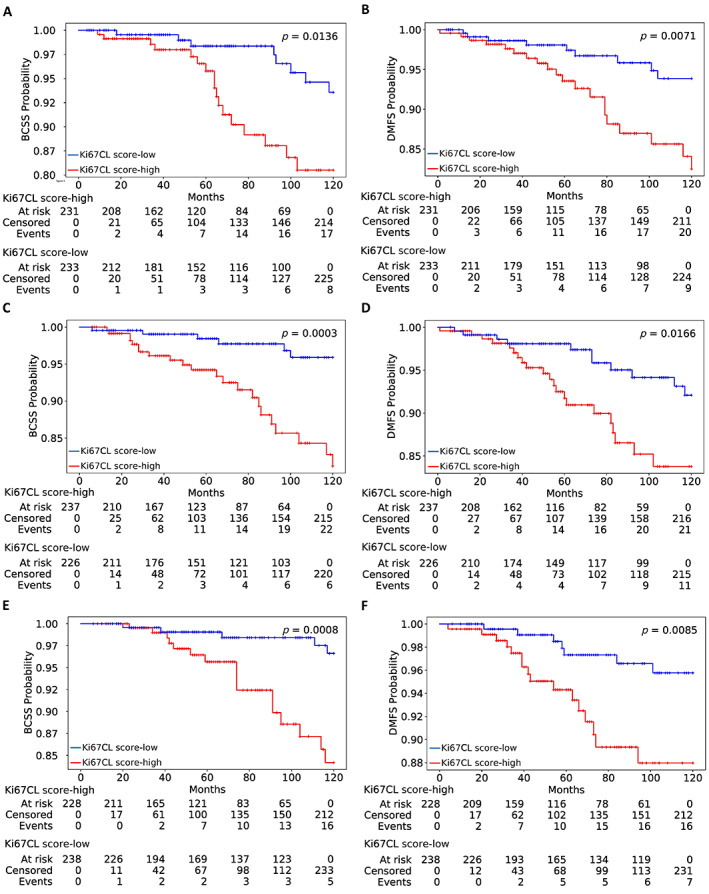
Kaplan–Meier curves of low‐risk (Ki67CL‐low) and high‐risk (Ki67CL‐high) patients stratified using the proposed marker Ki67CL score on three separate internal cross‐validation sets. Top to bottom (each row): cross‐validation folds 1–3. (A, C, E) BCSS; (B, D, F) DMFS.

The risk stratification performance using different markers on the validation sets of endocrine treated patients is listed in Table 1. Patients with high Ki67CL score had worse BCSS and DMFS than patients with low score (*p* = 0.00158, *C*‐index = 0.70). A similar *C*‐index (0.70) but less significant *p* value (0.01158) was achieved by AutoKi67 ratio. The Kaplan–Meier curves along with the corresponding *p* values on validation sets are presented in Figure 3. It is clear that Ki67CL score can stratify patients treated only with endocrine therapy into high‐risk and low‐risk groups significantly in all three validation sets.

**Table 1:** Prognostic significance of proposed Ki67CL score and AutoKi67 ratio for BCSS and DMFS

Feature	BCSS		DMFS	
	Combined *p* value	Combined *C*‐index	Combined *p* value	Combined *C*‐index
AutoKi67 ratio	0.01158	0.70 ± 0.008	0.02106	0.67 ± 0.021
Ki67CL score	0.00158	0.70 ± 0.009	0.01705	0.68 ± 0.005

**Figure 4:**
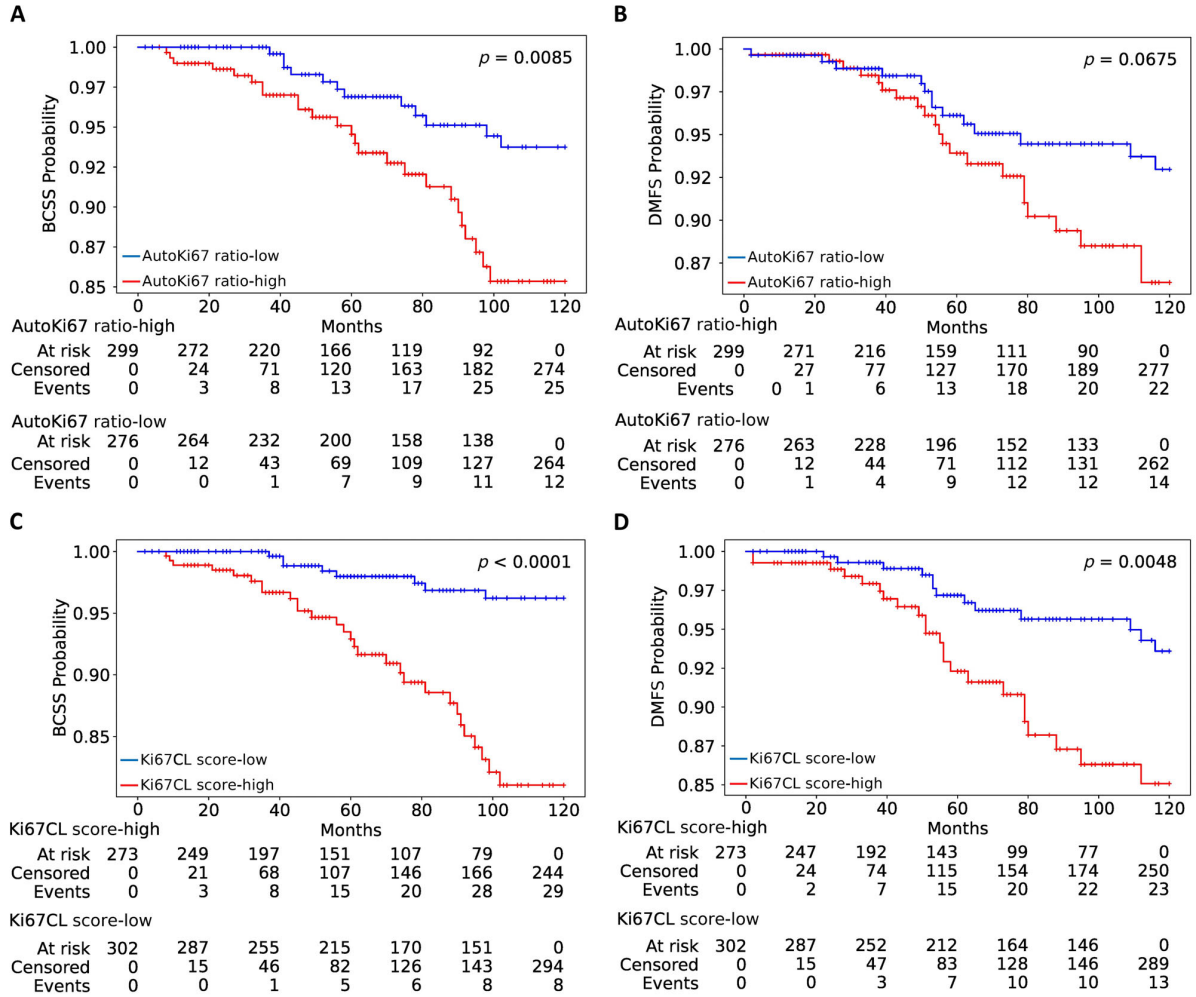
Kaplan–Meier curves of low‐risk (Ki67CL‐low) and high‐risk (Ki67CL‐high) patients stratified using different markers on the test set for BCSS (left column: A, C) and DMFS (right column: B, D). (A and B) AutoKi67 ratio, (C and D) Ki67CL score.

As both the Ki67CL and AutoKi67 scores achieved significant values on the validation cohorts, we tested both markers on the final test set and plotted the corresponding Kaplan–Meier curves (Figure 4). The survival plot curves show a clear separation between low‐ and high‐risk patient groups when stratified using the Ki67CL score (*p* < 0.0001, *C*‐index = 0.75 in BCSS and *p* = 0.004812, *C*‐index = 0.64 in DMFS). However, AutoKi67 score showed less significant separation in BCSS and achieved a lower *C*‐index in both BCSS and DMFS (*p* = 0.0085, *C*‐index = 0.71 in BCSS and *p* = 0.0675, *C*‐index = 0.61 in DMFS).

#### Effectiveness of adjuvant chemotherapy

**Figure 5:**
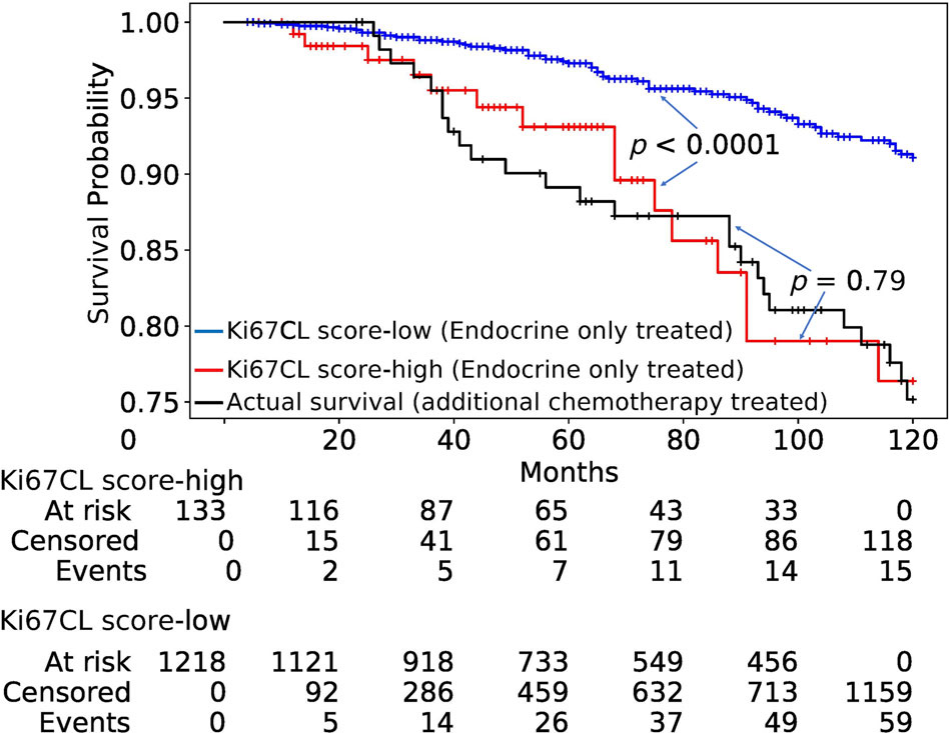
Kaplan–Meier curves of low‐risk (Ki67CL‐low) and high‐risk (Ki67CL‐high) patients treated only with endocrine therapy, stratified using the proposed Ki67CL score, and two control groups (no treatment and endocrine therapy with additional chemotherapy). There is no significant risk difference between our predicted high‐risk endocrine‐only treated cases and the cases who received both endocrine therapy and chemotherapy.

The proposed model was developed for patients who have been treated with endocrine therapy only and can stratify these patients into high‐ and low‐risk groups based on their survival probabilities as indicated by their Kaplan–Meier curves (blue and red) in Figure 5. These curves indicate that the two groups generated by the proposed model have statistically significantly different survival probabilities (*p* < 0.0001).

Our findings indicate that among the patients in our cohort who have received only endocrine therapy, the survival curve of the high‐risk group identified by the model is no different from that for patients who have received both endocrine therapy and additional chemotherapy (*p* = 0.79). This leads us to hypothesize that these high‐risk patients can potentially benefit from additional clinical therapy. However, this hypothesis needs further validation through a clinical trial but it does highlight the potential utility of the proposed approach to identify candidates for additional chemotherapy within patients who have only received endocrine therapy. Once backed up by further investigations and trials that the high‐risk cases identified by the proposed model do benefit from additional chemotherapy, the tool will be translated to clinical use to be used as an alternative to gene expression based tests currently in guidelines for this intermediate category of ER+/HER2− BC.

### Statistical independence of Ki67CL score from clinical factors and pathological variables

**Figure 6:**
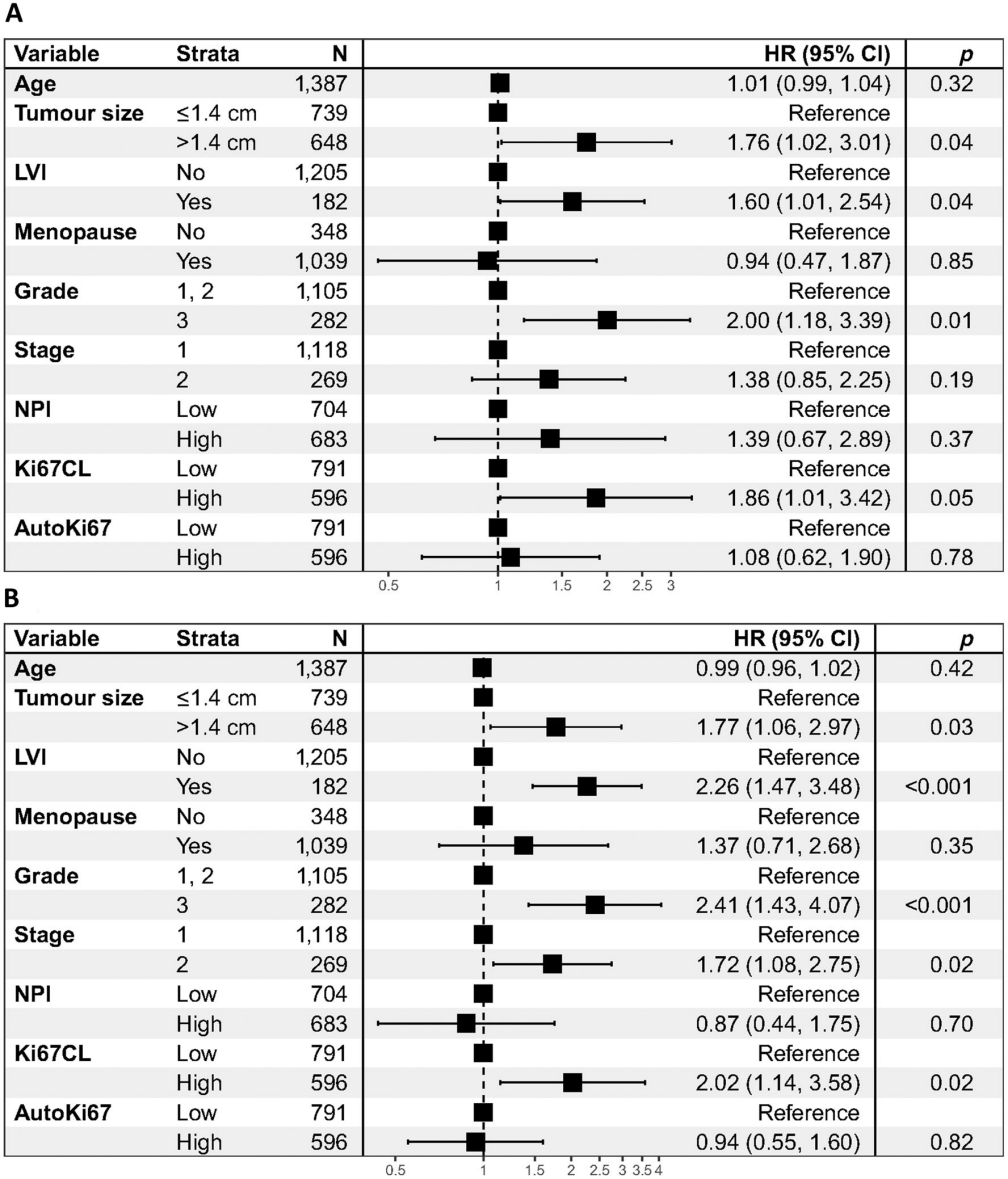
Multivariate analysis of Ki67CL score in the presence of available clinical and pathological variables. (A) BCSS; (B) DMFS.

We investigated the prognostic significance of Ki67CL score through multivariate analysis in the presence of clinical factors and pathological variables. It can be observed from Figure 6 that, for both BCSS and DMFS, Ki67CL score is an independent variable of statistical significance. In BCSS, taking the lower Ki67CL score group as the reference, the higher Ki67CL score group has a high hazard ratio of 1.86 (95% CI: 1.01–3.42) with a *p* value of 0.05. Similarly, for DMFS the group with higher Ki67CL score has a hazard ratio of 2.02 (95% CI: 1.14–3.58 in the high value group) with a *p* value of 0.02 compared to the group with lower Ki67CL score. It can be observed that the proposed marker Ki67CL score is positively correlated with the hazard ratio. A higher Ki67CL score value is associated with worse BCSS and DMFS.

#### Survival analysis with/without including DCIS regions

Our proposed feature Ki67CL score was extracted from the WSI level which may contain DCIS regions, while the compared AutoKi67 ratio was calculated from the invasive tumor region only. This is because conventional Ki67 scoring is carried out on the invasive tumor region [11, 12]. In this experiment, we extracted both features from two settings: WSI level (with DCIS) and invasive tumor region level (without DCIS), and evaluated their prognostic performance. DCIS regions were excluded by an Efficient‐UNet model [27] that was trained on pathologist annotated areas [regions of interest (ROIs)] in the discovery set. About 14,000 ROIs of size 1,024 × 1,024 pixels were used in a five‐fold cross‐validation to achieve F1 scores of 0.90 for DCIS and 0.71 for invasive tumor.

Table 2 shows the *p* values and *C*‐indices achieved by different features on the validation and testing sets. Ki67CL score performed slightly worse for BCSS in terms of *C*‐index when DCIS regions were excluded. The AutoKi67 ratio gives improved results when DCIS regions are excluded from the analysis, which is consistent with how conventional manual Ki67 scoring is performed. AutoKi67 ratio *C*‐index increases from 0.67 to 0.71 in BCSS and from 0.59 to 0.61 in DMFS on the test set. When DCIS regions are included, no significant prognostic performance is observed on the DMFS while using AutoKi67 ratio‐related features.

**Table 2:** Prognostic significance of features extracted from regions with/without DCIS regions

BCSS				
	Validation		Testing	
Feature	Combined *p* value	Combined *C*‐index	*p* value	*C*‐index
AutoKi67 ratio with DCIS	0.00672	0.69 ± 0.017	0.00851	0.67
AutoKi67 ratio without DCIS	0.01158	0.70 ± 0.008	0.00429	0.71
Ki67CL score with DCIS	0.00158	0.70 ± 0.009	<0.0001	0.75
Ki67CL score without DCIS	0.00005	0.70 ± 0.005	0.00051	0.73

DMFS				
	Validation		Testing	
Feature	Combined *p* value	Combined *C*‐index	*p* value	*C*‐index
AutoKi67 ratio with DCIS	0.05881	0.64 ± 0.033	0.067545	0.59
AutoKi67 ratio without DCIS	0.02106	0.67 ± 0.021	0.196384	0.61
Ki67CL score with DCIS	0.00040	0.68 ± 0.005	0.00481	0.64
Ki67CL score without DCIS	0.00023	0.68 ± 0.005	0.042189	0.63

#### Ki67CL score visualization

The score for spatial colocalization between Ki67‐positive tumor and Ki67‐negative tumor cells in each cluster was computed using Equation (1). Then, the overall Ki67CL score at WSI level was computed by averaging the cluster‐level scores and used for the survival analysis. Supplementary material, Figure S4A shows some example regions of low, medium, and high Ki67CL score while supplementary material, Figure S4B gives visualization of Ki67CL score quantification at the WSI level and the region level.

## Discussion

The majority of ER+/HER2− BC patients belong to an intermediate risk group in which some of the patients may benefit from adjuvant chemotherapy in addition to endocrine therapy [28]. Even though several risk calculators have been developed to estimate a patient's risk of BC recurrence and mortality, these methods are expensive and have long turnaround times. Ki67 is a proliferation marker in BC that has been proposed as a useful clinical marker for BC prognosis and prediction of therapeutic response [12, 20]. However, Ki67 scoring shows poor reproducibility due to lack of standardization across pathologists and laboratories [13].

In this study, we developed an AI‐based tool to extract a Ki67‐based risk score, named Ki67CL score, for risk stratification of intermediate risk BC patients who were treated with endocrine therapy only. We found that Ki67CL score can categorize patients into different risk groups; low Ki67CL (≤0.375) as low‐risk group and high Ki67CL (>0.375) as high‐risk group. Also, a significant association between high Ki67CL score and tumor characteristics associated with aggressive tumor behavior was observed indicating that this score can be used as a surrogate marker in luminal BC.

In comparison with AutoKi67 ratio, Ki67CL score showed more significant risk stratification of luminal BC patients both in terms of DMFS (*p* values 0.0211 and 0.0004, respectively) and BCSS (*p* values 0.0116 and 0.0016, respectively). Based on multivariate analysis, Ki67CL score is an independent prognostic marker and adds information above the clinical and pathological variables and AutoKi67 ratio in the current study cohort. This suggests the importance of the colocalization of Ki67‐positive and Ki67‐negative tumor cells. Similarly, the association of Ki67CL score and AutoKi67 ratio with other clinicopathological variables shows that Ki67CL score is more significantly associated with histologic tumor grade, NPI, and LVI than AutoKi67 ratio.

Ki67CL stratified endocrine‐treated patients into two distinct prognostic groups. However, there was no significant difference between high‐risk patients classified by Ki67CL score and the group of patients who received additional chemotherapy. After validation on a larger cohort and further clinical trials, the proposed risk score might help make the decision on whether to prescribe adjuvant chemotherapy. For both BCSS and DMFS, Ki67CL score was an independent variable of statistical significance. High Ki67CL was also associated with high hazard risk which indicates that a higher value of the Ki67CL score is associated with poorer patient survival and greater liability for recurrence.

Lack of standardization of manual Ki67 scoring encouraged the search for automated Ki67 scoring methods like DIA [14]. However, standardization and subsequent clinical validation are still required. To the best of our knowledge, our proposed Ki67CL score is the first Ki67‐based prognostic marker which stratifies patients' risk by measuring the spatial colocalization between Ki67 tumor cells. From the analysis comparison, this colocalization‐based score is also shown to be more significant and more stable for risk stratification than the popularly used Ki67 score.

The predictiveness of the proposed Ki67CL score was demonstrated in a large cohort but the study still has some limitations. The information added by the proposed score above clinical and pathological variables needs to be further corroborated by multivariate analysis in other larger cohorts. Similarly, the survival curve of the endocrine therapy only high‐risk group predicted by the proposed score was very much similar to the group in which additional chemotherapy was administered but this needs to be further validated in a larger control group. Furthermore, a single slide per case was used by the ML model to make a prediction but to cover more heterogeneity multiple slides per case can also be utilized. For better reproducibility of the proposed score, it is also important to validate and perhaps also train on multicentric data.

Ki67‐positive/negative tumor carries useful information regarding prognosis but the inclusion of other features from different immune cells, such as CD4 and CD8 positive cells, and interaction of immune cells with Ki67‐positive/negative tumor cells merit investigation in future work. To make the Ki67‐based analysis more robust, stain intensity might also be used to reduce the noise in pathologists' annotations by discarding positive cell below a certain threshold. In clinical practice, both H&E and biomarker information are used as prognostic and predictive indicators. Combining the proposed Ki67‐based prognostic marker with features extracted from H&E staining and clinical factors may have the potential to achieve better risk stratification performance. We hope this work will encourage further research in image‐based prognostics in breast and other types of cancer.

## Author contributions statement

NR, ER and WL conceived the study. WL performed experiments. MJ and SG carried out tumor segmentation. MT, IMM, AGL, NMA, SM and AYI performed annotation. MT, ER, IMM, SEAR, DS and AB collected data. WL and MB performed image quality control. NW, NR, SEAR and FM wrote and edited the paper. AGL, NMA, AB, ER and SM reviewed the paper. All authors read and approved the final paper.

## Supporting information


**Figure S1.** Examples of Ki67‐positive and Ki67‐negative tumor and nontumor cells
**Figure S2.** Illustration of Ki67CL score (Ki67‐positive tumor and Ki67‐negative tumor co‐localization pattern)
**Figure S3.** Cell detection and classification performance on sample test images
**Figure S4.** Ki67CL score visualization
**Table S1.** Number of cases in discovery, validation, and testing cohorts
**Table S2.** Patient and tumor characteristics
**Table S3.** Cell classification results of the proposed model for individual cell classes
**Table S4.** Relationship between Ki67 scores (Ki67CL score and AutoKi67 ratio) and other clinicopathological variables

## Data Availability

All annotations and the corresponding data will be made available upon completion of the PathLAKE project.
