# Supplementary material for: AI‐based intra‐tumor heterogeneity score of Ki67 expression as a prognostic marker for early‐stage ER+/HER2− breast cancer
Source: J Pathol Clin Res. 2023 Oct 24;10(1):e346. doi: 10.1002/cjp2.346 (PMC10766021; doi:10.1002/cjp2.346)
Supplement: Supplementary file 1 — Figure S1. Examples of Ki67‐positive and Ki67‐negative tumor and nontumor cells Figure S2. Illustration of Ki67CL score (Ki67‐positive tumor and Ki67‐negative tumor co‐localization pattern) Figure S3. Cell detection and classification performance on sample test images Figure S4. Ki67CL score visualization Table S1. Number of cases in discovery, validation, and testing cohorts Table S2. Patient and tumor characteristics Table S3. Cell classification results of the proposed model for individual cell classes Table S4. Relationship between Ki67 scores (Ki67CL score and AutoKi67 ratio) and other clinicopathological variables [file CJP2-10-e346-s001.pdf]

AI-based intra-tumor heterogeneity score of Ki67 expression as a prognostic marker for early-stage ER+/HER2− breast cancer

W Lu *et al. J Pathol Clin Res* https://doi.org/10.1002/cjp2.346

Supplementary Figures S1–S4
Supplementary Tables S1–S4

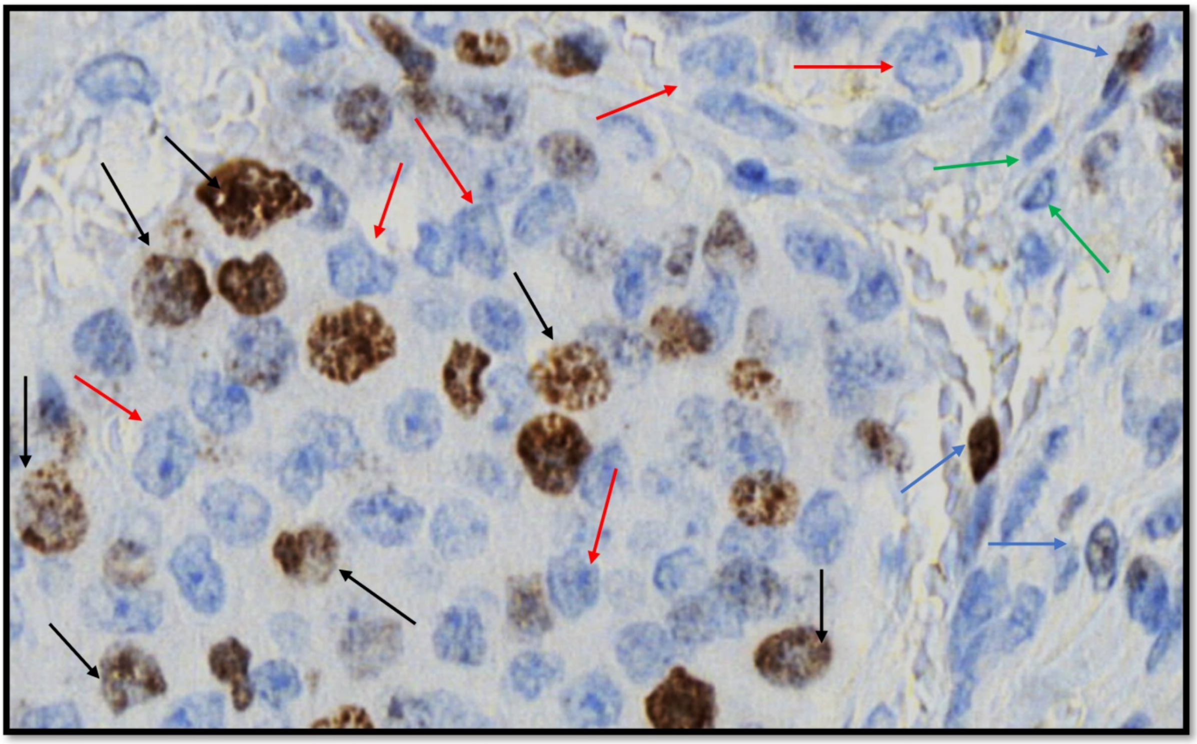

Figure S1. Examples of cell types at ×40. Ki67 positive tumor cells (black arrows); Ki67 negative tumor cells (red arrows); Ki67 positive nontumor cells (blue arrows); Ki67 negative nontumor cells (green arrows).

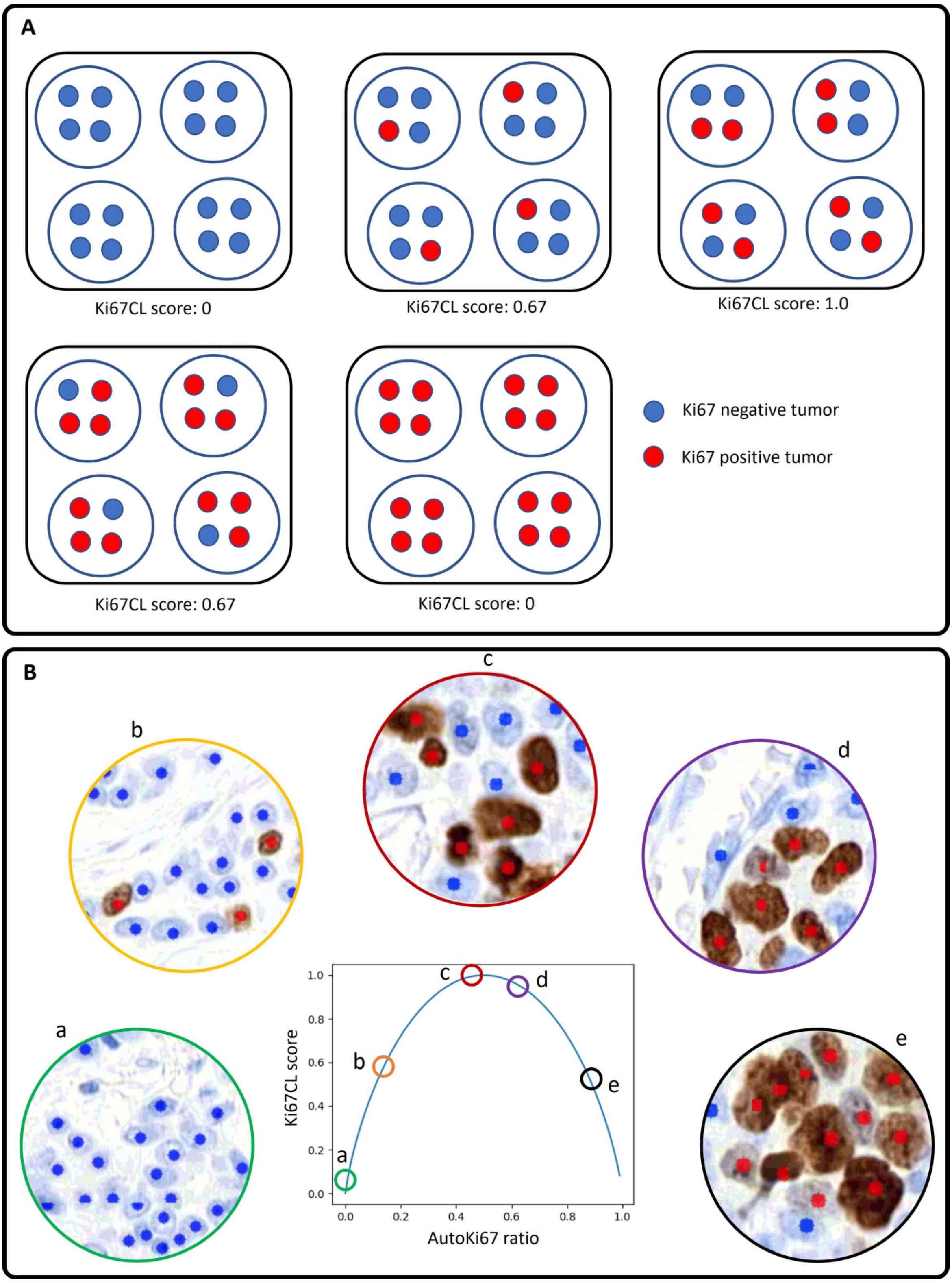

Figure S2. Illustration of Ki67CL score (Ki67 positive tumor and negative tumor co-localization pattern). (A) Each black square represents one WSI and each black circle inside represents one cluster of Ki67 positive and negative tumor cells. (B) Ki67CL score varies when AutoKi67 ratio i.e., percentage of Ki67 positive tumor cells changes from 0 to 100%. Five clusters with different Ki67 positive tumor cells ratio are given for feature illustration.

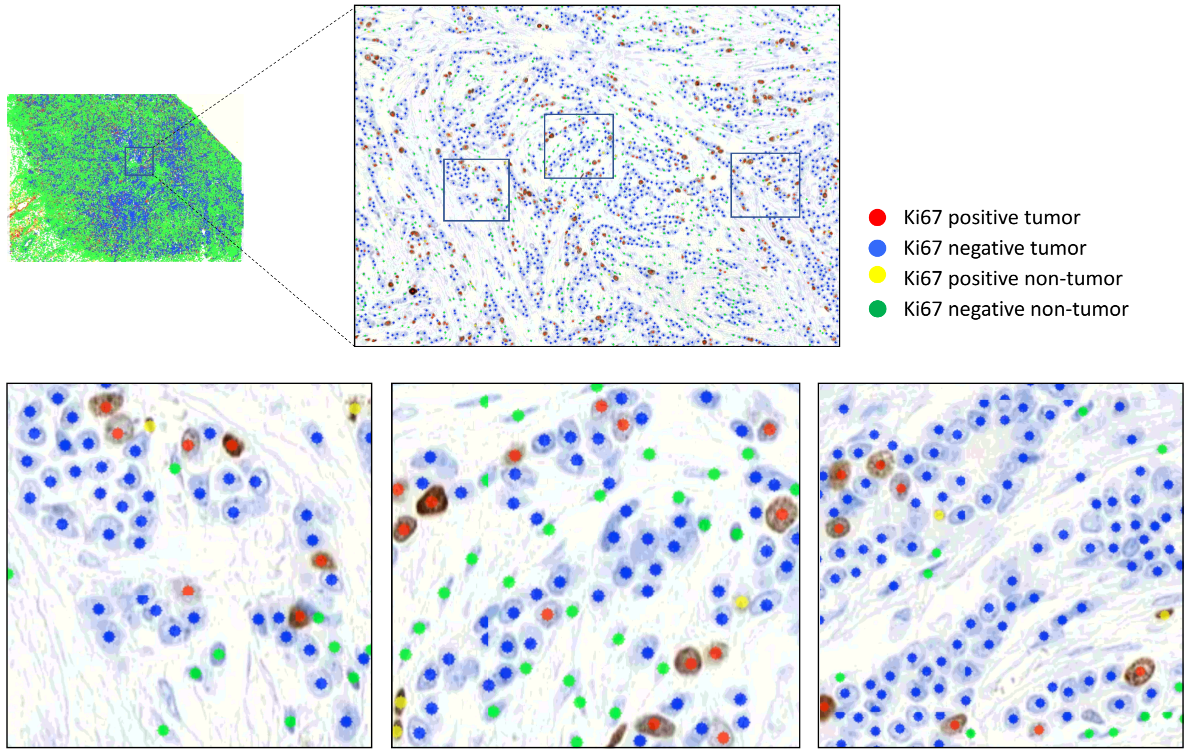

Figure S3. Cell detection and classification performance on sample test images. Red: Ki67 positive tumor; Blue: Ki67 negative tumor; Yellow: Ki67 positive nontumor; Green: Ki67 negative nontumor.

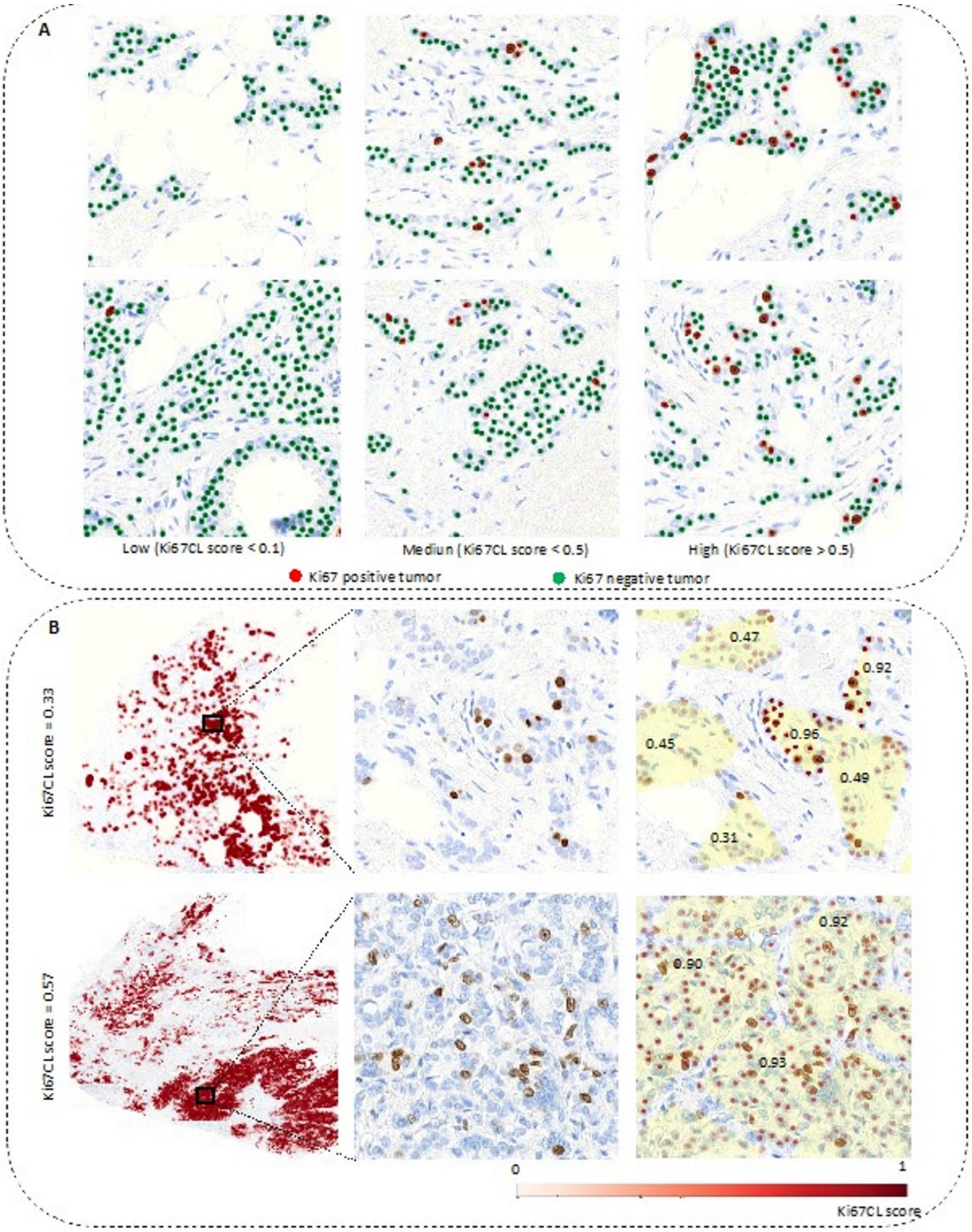

Figure S4. Ki67CL score visualization. (A) Example regions of low, medium and high Ki67CL score (red: positive tumor, green: negative tumor). (B) Visualization of Ki67CL score quantification on the WSI-level and region-level. Each yellow region represents a cluster and its corresponding Ki67CL score value is attached.

Table S1. Number of cases in discovery, validation and testing cohorts

Fold	No. in discovery cohort	No. in validation cohort	No. in testing cohort
**1**	930	463	/
**2**	930	463	/
**3**	927	466	/
**Test**	/	/	685

Table S2. Patient and tumor characteristics. SD=standard deviation

Variable		Number	%
**Patient age at diagnosis (years)**	Mean (SD)	60 (11.55)	-
	Range	20-92	-
**Nottingham histological grade**	Grade 1	468	22.52
	Grade 2	1180	56.79
	Grade 3	430	20.69
**Nuclear pleomorphic score**	1	13	0.63
	2	1048	50.43
	3	1017	48.94
**Tubule formation score**	1	217	10.44
	2	635	30.56
	3	1226	59.00
**Mitotic score**	1	1496	71.99
	2	315	15.16
	3	267	12.85
**Tumor size (cm)**	Mean (SD)	1.72 (0.96)	-
	Range	0.15-10.0	-
**Positive lymph nodes**	0	1631	78.49
	1-3	447	21.51
**Histological tumor type**	Not otherwise specified (NST)	1179	56.74
	Lobular	259	12.46
	Other special types	131	6.30
	NST mixed	509	24.49
**Nottingham Prognostic Index (NPI)**	Good (≤3.4)	1170	56.30
	Moderate (≥3.5 and ≤5.4)	851	40.95
	Poor (>5.4)	57	2.74

Table S3. Cell classification results of the proposed model for individual cell classes.

Cell type	Kappa score	Spearman correlation	Accuracy	F1-Score
**Positive tumor**	0.81	0.82	0.97	0.83
**Negative tumor**	0.77	0.78	0.85	0.87
**Positive nontumor**	0.71	0.72	0.96	0.75
**Negative nontumor**	0.74	0.75	0.86	0.78

Table S4. Relationship between Ki67 scores (Ki67CL score and AutoKi67 ratio) and other clinicopathological variables.

	**Mann-Whitney *U* test (*p* value)**	
**Categories**	**Ki67CL score**	**AutoKi67 ratio**
**Tumor size**<br>≤2 cm vs >2 cm	1.0	0.870
**Tumor grade**		
Grade 1 vs 2	6.497e-19	2.033e-10
Grade 1 vs 3	4.531e-52	7.160e-30
Grade 2 vs 3	5.895e-36	1.326e-17
**Lymph node status**<br>Positive vs Negative	0. 922	0.999
**Lymphovascular invasion**<br>Present vs Absent	0.0090	0.8322
**NPI**		
Good vs Moderate	5.454e-29	8.776e-09
Good vs Poor	2.480e-06	0.0365
Moderate vs Poor	0.0123	0.0972
